# Delivering Bad News: Self-Assessment and Educational Preferences of Medical Students

**DOI:** 10.3390/ijerph19052622

**Published:** 2022-02-24

**Authors:** Julia Lenkiewicz, Oliwia Lenkiewicz, Marcin Trzciński, Krzysztof Sobczak, Jan Plenikowski, Julia Przeniosło, Agata Kotłowska

**Affiliations:** 1 Faculty of Medicine, Student Scientific Circle of Medical Communication at the Medical University of Gdansk, 80-210 Gdansk, Poland; julialenkiewicz@gumed.edu.pl (J.L.); oliwialenkiewicz@gumed.edu.pl (O.L.); m.trzcinski@gumed.edu.pl (M.T.); jan-plenikowski@gumed.edu.pl (J.P.); julia.przenioslo@gumed.edu.pl (J.P.); agatakotlowska@gumed.edu.pl (A.K.); 2 Department of Sociology of Medicine and Social Pathology, Medical University of Gdansk, 80-210 Gdansk, Poland

**Keywords:** delivering bad news, diagnosis, truth disclosure, doctor-patient relationship, medical communication

## Abstract

Background: Numerous reports indicate the educational deficiencies of medical students in delivering bad-news-related skills. Evaluation of the performance of training programs in this area should be one of the key components of the educational process. The purpose of this study was to analyze medical students’ preferences and educational needs regarding DBN (delivering bad news). The effect of clinical experience on the self-assessment of skills was analyzed. Methods: The quantitative survey was conducted using the CAWI technique. The study involved 321 fifth- and sixth-year medical students from 14 medical universities in Poland. Pearson’s χ^2^ test was used for statistical analysis. The profile of respondents for categorical variables was determined by KMeans analysis. Results: As many as 75.1% of students revealed that they did not feel sufficiently prepared for DBN. Only 18.7% reported having adequate competence in this area. More than half of the inquired students (63.6%) witnessed a situation during their clinical practice in which a physician provided a patient with information about an unfavorable diagnosis. These students were less likely to declare that they could not deliver BN (43.4%) than students who had no such experience (58.2%). As many as 86.3% of the respondents reported the need for more time in DBN skills training. Students mostly preferred active teaching methods. Conclusions: Understanding students’ learning needs and preferences can help medical schools optimize their education programs to develop DBN-related competencies.

## 1. Introduction

Delivering bad news (DBN) is one of the most difficult challenges a physician faces [1]. This task is often accompanied by difficult emotions, which is associated with experiencing a high psychophysical burden [2]. As indicated by the results of studies, physicians’ attitudes and behaviors in DBN situations correlate with patients’ decisions to discontinue or continue treatment [3]. The way the unfavorable message is delivered affects the level of patients’ motivation to continue treatment and translates into therapeutic outcomes [3]. Therefore, proper teaching of soft skills is very important in the process of medical education.

Medical schools are increasingly introducing courses to develop skills necessary for situations of unfavorable medical information delivery [4,5]. Many analyses have revealed the educational effectiveness of training provided in this area [6,7]. However, still not enough students in the course of their education receive formal training developing skills related to DBN [8,9]. In their international study, A. Alshami et al. showed that only 26.6% of medical students received formal DBN training during their education [10]. The deficit of this type of instruction causes students to still report the need for in-depth training in this area [11,12]. Although competency development courses in delivering bad news appear to be relatively effective, they may not meet all expectations. Multiple research studies reveal that the implementation of skills obtained during the DBN courses in practice constitutes a significant challenge. The reason behind this problem is that the conversations between the patient (usually played by an actor or another student) and the future doctor are performed in artificial and controlled environments. Therefore, students do not get the chance to practice their DBN skills in real-life situations [13,14].

There are 21 universities in Poland that offer undergraduate courses in Medicine. All of these educational institutions teach a 12-term (6-year) Medicine course that ends with an MSc in Medicine [15]. The subjects taught during the first six terms (three years) are mainly theoretical, while clinical subjects are taught from the fourth year until the sixth year of the course. The courses that form DBN skills are mostly optional or are not offered at all. There is some information on how to deliver BN on different subjects, such as Psychology or Oncology, but it mainly depends on the assistant if the topic of DBN is discussed during classes. The vast majority of medical students in Poland obtain their communication skills through clinical practice with no prior formal training.

There is a significant shortage of studies reporting students’ self-assessment of DBN preparation in the context of their own clinical experiences. Our research questions were the following: (1) How do fifth- and sixth-year medical students self-assess their preparedness for DBN? (2) What are the educational needs and preferences of fifth- and sixth-year medical students regarding DBN-related skills training? We wanted to find out how clinical experience verifies students’ self-assessment of their competence in the area of communicating an unfavorable diagnosis and what students’ educational expectations are in this area. We assumed that unfavorable diagnosis would refer to all information about the diagnosis of a disease that is associated with changes in the body requiring permanent or long-term treatment or therapy aimed at symptom relief and pain management. Understood in this manner, it refers to civilization diseases (e.g., diabetes, coronary heart disease, allergies, cancer, etc.), as well as psychiatric, genetic, and incurable fatal diseases [3].

## 2. Materials and Methods

### 2.1. Study Design

The research was a cross-sectional study. To obtain data, we used the quantitative correlational method and the CAWI technique (Computer-Assisted Web Interview). We prepared an original research tool, which was submitted for approval to the Independent Bioethics Committee for Scientific Research of the Medical University of Gdansk (NKBBN/287/2021). The subject of our interest was students’ experiences and their expectations related to DBN competence training. In this report, we focused on presenting the results regarding the experiences and needs revealed by the respondents in DBN-related contexts.

### 2.2. Setting

The field research was conducted between 15 April and 1 July 2021. The e-questionnaire was made available on a service dedicated to conducting professional sociological research (www.ebadania.pl, accessed on 1 July 2021). Respondents were provided with full information about the purpose and conduct of the study before taking part. Participation in the study was completely voluntary and anonymous, and no sensitive data or data that could identify students were collected or processed during the study. Each participant confirmed their voluntary consent to participate in the study. Because of the pandemic, we recruited study participants using medical school newsletters, mailing lists, and social media. We sent invitations to participate in the study to the deans’ offices and other administrative structures of medical universities across the country.

### 2.3. Participants

We addressed our invitation to participate in the study to fifth- and sixth-year medical students. As many as 321 students from 14 medical schools across the country participated in the study. All responses obtained from respondents were included in the analysis.

In the academic year 2020/2021, there were 21 universities in Poland offering an undergraduate course in Medicine to approximately 34,000 students. However, the official government statistics do not offer information about the exact number of students in the fifth and sixth year of their studies. Therefore, we were not able to determine the quota sampling for the study group, and it should be assumed that the study group in our research is not representative.

### 2.4. Variables

We compared the quantitative data with the obtained continuous variables, analyzing the presence of significant statistical differences and correlations between them. We analyzed associations between gender, year of study, preference for choice of future specialty, and declared level of experience with patients other than mandatory clinical practice. We considered the personal experience of receiving unfavorable medical news from a physician as an independent variable. The continuous variables analyzed for this article involved seven closed-ended questions. These were related to students’ experiences (witnessing the situation in which the physician delivers bad news, being a recipient of BN, being a deliverer of BN), preferred methods of competency development (need for increased time for skills training related to communicating difficult medical information during mandatory courses, opinion on the teaching methods that students think would be effective in developing their communication skills), and self-reported feelings of preparedness for DBN situations (sense of preparation for DBN, concerns about the situation of DBN). For questions for which we used a Likert scale, we categorized “definitely yes” and “rather yes” and “rather no” and “definitely no” responses as affirmative and negative cafeterias.

### 2.5. Statistical Methods

Statistical analysis was performed using Statistica v. 13. We used Pearson’s χ^2^ test to analyze relationships between discontinuous variables and statistical heterogeneity between groups. The difference was considered statistically significant for *p* < 0.05. In determining the profile of the subjects for categorical variables, we used KMeans analysis.

## 3. Results

The study included 321 fifth- and sixth-year medical students from 14 national medical schools (Table 1). We obtained an overrepresentation of the female group in the study. According to the Central Statistical Office, in the 2018/2019 academic year, the percentage of female medical students in Poland was 59.6% [16]. Almost 35% of all students who participated in the study studied at the Medical University of Gdansk.

### 3.1. Self-Assessment of Preparedness

The vast majority (75.1%, *n* = 241) of participants revealed that they did not feel that medical school prepared them well enough for DBN-related tasks. Only 18.7% (*n* = 60) felt that they received such competencies during their studies. Twenty students (6.2%) did not answer our question.

We wanted to know if students during their clinical placements witnessed a situation in which they could observe a physician communicating an unfavorable diagnosis to a patient. Exactly 36.4% (*n* = 117) admitted that they had no such experience, but the majority revealed (63.6%, *n* = 204) that they had participated in observing a physician during notification. Of this group, only 11.8% (*n* = 24) considered this situation to lack educational value; 14.2% (*n* = 29) could not qualify its impact on the development of their own competence. However, the vast majority (74%, *n* = 151) of students found the experience informative.

Based on the analyses of the study group, we found significant statistical differences between the experience of observing situations in which doctors provide unfavorable medical information to patients and the self-assessment of their competence in this area. Students who had not witnessed such situations were more likely (58.2%) to declare that they could not provide information about unfavorable diagnosis than those who had such educational experience (43.4%; statistic: χ^2^ = 4.303; df = 1; *p* = 0.038).

### 3.2. Feedback on Educational Needs

Only 13.7% (*n* = 44) students felt that the time devoted to skills training in the area of DBN was sufficient. As many as 86.3% (*n* = 277) declared the need to increase it. Such positions were more often held by women (90.1%) than men (77%; statistic: χ2 = 9.274; df = 1; *p* = 0.002). In addition, students who had additional experience with patients beyond mandatory clinical practice (90.1%) were more likely to declare the need for more time for DBN education than others (80.6%; statistic: χ^2^ = 5.867; df = 1; *p* = 0.015).

### 3.3. Choice of Specialty vs. Preference

More than half (57.6%) of the students we interviewed revealed that they associate the development of their medical career with the choice of a non-surgical specialty. This criterion proved to be an important variable associated with feeling prepared for DBN. Students who intend to choose a non-surgical specialty were most likely (89.7%) to report the need for more educational time related to developing DBN competencies (77.2%; statistic: χ^2^ = 7.195; df = 1; *p* = 0.007). They were more likely (55%) than those choosing treatment specialties (32.7%) to report a lack of skills necessary to communicate an adverse diagnosis (statistic: χ^2^ = 7.230; df = 1; *p* = 0.007). They were also more often (73.2%, *n* = 131) concerned about patients’ emotional reactions as a response to the news of an unfavorable diagnosis (52%; statistic: χ^2^ = 14.885; df = 1; *p* < 0.001).

### 3.4. Preferences in Terms of Teaching Methods

Future physicians most often preferred active learning methods in their choices, through which they could be indirectly immersed in the DBN experience. They also frequently indicated passive methods that could expose them to the dynamics of the doctor-patient relationship and the complexity of these interactions. They were least likely to favor other types of passive methods that emphasized knowledge development rather than skill development (Table 2).

Through KMeans analysis, we profiled the preferred teaching methods (Table 3). Almost half of the students preferred learning methods based on role-playing with a simulated patient and DBN exercises with ongoing feedback from an observing psychologist. Individuals in this group did not indicate other forms of education as effective in developing their competence. The second group of students simultaneously preferred watching videos presenting the most common mistakes and showing the best methods of DBN and exercises with active participation of the psychologist. The third group of students preferred all methods of education. The last group, which was the least numerous, preferred only the independent study of literature.

## 4. Discussion

A self-assessment study of students’ sense of preparation for DBN can be considered as one of the methods of evaluating the effectiveness of medical preparation training. Using an anonymous and structured questionnaire, we were able to find out the concerns and emotions of the respondents, as well as to determine the level of their sense of preparedness for real clinical situations. The analysis of students’ experiences is important for strengthening the effectiveness of educational models. Evaluation of the performance of DBN training programs should be one of the key components of the educational process in medical universities [17].

Our survey revealed that the majority of students (75.1%) felt inadequately prepared to deliver bad news. Only 18.7% (*n* = 60) of those asked felt that they had acquired sufficient competence in this area during their studies. The level of stratification of the self-assessment scale of preparation for BN notification seems to be a relatively common phenomenon. This observation was also confirmed in the study by L. A. Díaz-Martínez et al. In a group of 438 students, 64.1% of the respondents admitted that they did not feel well prepared for DBN tasks. Only 10.5% (*n* = 46) of the students indicated that their skills in this area were sufficient [18]. In addition, a study by E. F. de Moura Villela et al. showed that there are gaps in DBN education in the medical school curriculum. As many as 90% of the students surveyed admitted that they had never received training in DBN [8]. The similarities in the results of the above studies may indicate a deficit of BN delivery classes in curricula.

The majority (63.6%) of those asked revealed that they had the opportunity to observe physicians delivering BN during their clinical practice. Exactly 74% of these respondents found this type of situation to be an evolving learning experience. It seems that such experience can be an important part of educating future physicians. An analogous observation was noted by J. V. Kiluk et al. among students completing an interdisciplinary oncology internship. In the analyzed group of students, as many as 95.5% had a chance to be DBN observers during their medical school education. As many as 63.4% of the students revealed that they felt “extremely comfortable/somewhat comfortable” in this situation [19]. In our study, we noticed a connection confirming this relationship. Students who observed the behavior of physicians and patients during the DBN were less likely to agree with the statement that they were unable to communicate unfavorable medical information. It seems to us that immersion in this type of experience allows one to better understand one’s own emotions, as well as the difficult experiences of patients. Observing DBN may provide students with an experience where they gain the opportunity to develop strategies for managing difficult emotions [20]. The additional opportunity to confront their observations and discuss them with the physician making the notification can provide an important educational platform.

When asked about the preferred types of training that can effectively develop DBN competencies, the largest number of students (46.7%) indicated active methods where immersion in the role of being a BN provider becomes possible. In our study, the largest group (80%) of students were interested in practicing conversations that a psychologist would observe and provide ongoing feedback. A total of 57% preferred exercises that involved role-playing with a simulated patient. Evaluations of courses that develop communication skills show that students preferred active forms of instruction. Exercises with a standardized patient are those that significantly increase communication skills [21]. Similar conclusions are drawn from a study by M. E. Rosenbaum et al., which indicated the greatest effectiveness of classes that include activities such as exercises with a standardized patient, group discussion, and role-playing, in which the student can take on the role of not only a physician but also a patient [6]. J. V. Kiluk et al. showed that as many as 98% of students found the activities with a standardized patient helpful. Watching recorded videos of situations simulating DBN by yourself or another student and discussing them also proved to be a useful experience [19]. In all of the above studies, students demonstrate the need to immerse themselves in an authentic patient interview experience. In this way, they are able to realistically test their communication skills and identify their own deficits in this area. It is evident from the above studies that active learning methods, through the perspective of immersing students in the roles of doctors and patients, provide opportunities to deepen their experiences. They develop decentration and empathy.

Among the students we surveyed, as many as 86.3% of them expected an increase in the amount of time devoted to teaching DBN competencies. This opinion was most often presented by three groups: women, students who have additional experience working with patients, and those who intend to choose non-surgical specialties. Medical students seem to be aware of the deficits in their DBN skills. They commonly report the need for more educational hours on developing DBN competencies. It is interesting to note that this type of preference is reported regardless of the educational systems pursued. A. H. Douglas et al., in their study, showed that most medical students in Nepal report a need for in-depth DBN education [9]. In a study by L. A. Díaz-Martínez, only 3.3% of Colombian students reported that they would not be interested in a course to improve competence in DBN [18]. In addition, a Chinese study revealed that the implementation of additional courses in this area is needed [22]. In Germany, in a study by A. Simmenroth-Nayda et al., students declared the need for more time to improve DBN skills [23]. Although the above studies were conducted in different countries, and the students had different ethnic and cultural proclivities, they have similar conclusions. We can think that the problem of inadequate DBN training is a relatively common case that still seems to not be taken into account when creating educational plans for medical students.

The decision to choose the type of specialty significantly correlated in our study with the declaration of the need to increase the time of communication skills training. Physicians choosing surgical specialties usually have limited contact with patients and thus show less interest in developing soft skills. In her study, M. M. Barnett showed that surgical physicians perform worse in conversations about an unfavorable diagnosis [24]. Similar findings were found in the study by K. Sobczak et. al., where differences in communication styles of physicians working in surgical and conservative departments were observed. The physicians with surgical specialties used open questions less frequently and were less interested in whether all the messages conveyed by them were understood by the patient. They also provided less detailed feedback to patients than physicians in conservative departments [25]. In our study, we revealed that students who intend to choose a surgical specialty are less likely to report a need for more time in communication skills training and a lack of adequate DBN competencies. Students declaring to choose a surgical specialty are less likely to announce that they are concerned about patients’ emotional reactions during DBN. Comparing these data, we can see that the motivations for developing DBN skills among those choosing surgical and non-surgical specialties are formed already at the stage of education. Students choosing surgical specialties, declare less enthusiasm for learning and developing DBN skills, which may have implications for less effective communication with patients after graduation.

The study we conducted has several strengths, but we are also aware of several objective limitations that occurred during the implementation of the study. First, the density distribution analysis revealed an overrepresentation of students from the Medical University of Gdansk. All those who participated in the study attended Polish universities. Given these parameters, caution should be taken when comparing results obtained by researchers from other countries. An additional element affecting the interpretation of the results are differences in educational programs. DBN education is specific. Competency-building courses are either mandatory or optional, and at some universities, they do not exist at all. These are variables that can modify students’ cognitive and emotional skills across universities and countries. The only form of education in which all medical students participate is learning through clinical practice. However, the organization of education in this area also varies. Teaching methods may significantly condition students’ opportunities to observe or provide BN during clinical placements. Our study was based on students’ self-assessment. Thus, it may result in retrospective error. Moreover, our study is not representative. We were not able to obtain information about the sample size. Thus, we could not assess the precise number of fifth- and sixth-year medical students in Poland.

## 5. Conclusions

Our results indicate that the real-world immersion associated with the DBN experience increases students’ sense of preparedness for the task. Devoting more time and modifying educational programs by introducing active learning methods could positively affect medical students’ overall competence. It is worthwhile, when planning educational interventions related to DBN, to take into account students’ preferences for choosing a future specialty.

The results of our study may help academic and clinical educators to better design the content and forms of education. Understanding students’ learning needs and preferences can contribute to optimizing educational courses so that they develop students’ competencies in patient communication and enable complete immersion in the DBN experience. The implementation of active learning methods (role-playing, SP) could improve students’ engagement as well as the effectiveness of DBN education.

## Figures and Tables

**Table 1:** Characteristics of respondents.

Variable	*n* = 321
	*n* (%)
Gender	
Female	232 (72.3)
Male	87 (27.1)
Age	
23–24 years	152 (47.4)
25 years or more	169 (52.6)
Year of study	
Fifth year	165 (51.4)
Sixth year	156 (48.6)
Experience in dealing with patients *	
Physician Assistant	44 (13.7)
Volunteering	26 (8.1)
Work in academic societies	66 (20.6)
Other than listed	56 (17.4)
Lack of experience	129 (40.2)
Medical schools	
Medical University of Gdansk	112 (34.9)
Medical University of Warsaw	38 (11.8)
Medical University of Lodz	34 (10.6)
Collegium Medicum Jagiellonian University	30 (9.3)
Medical University of Wroclaw	23 (7.2)
Collegium Medicum of Nicolaus	18 (5.6)
Copernicus University	
Pomeranian Medical University in Szczecin	16 (5)
Medical University of Poznan	13 (4)
Medical University of Silesia	11 (3.4)
Other	26 (8.1)
Preferred type of future specialty	
Non-surgical	185 (57.6)
Surgical	79 (24.6)
In the course of decision	57 (17.8)

* outside of mandatory clinical placements.

**Table 2:** Student opinions on preferred DBN teaching methods with significance for cluster analysis (*n* = 321).

Educational Methods *	*n* (%)	df	χ^2^	*p*	G^2^	*p*
Role-playing with a simulated patient	183 (57)	3	68.987	<0.001	75.190	<0.001
Watching videos showing the most common mistakes	177 (55.1)	3	167.959	<0.001	192.606	<0.001
Watching videos showing the best ways to DBN	181 (56.4)	3	179.048	<0.001	207.988	<0.001
Lectures	70 (21.8)	3	75.598	<0.001	63.171	<0.001
Participation in training led by a psychologist who provides ongoing feedback	257 (80)	3	38.500	<0.001	31.610	<0.001
Independent literature study	45 (14)	3	146.273	<0.001	102.083	<0.001

* respondents were allowed to indicate more than one answer.

**Table 3:** Preference of choice of education methods for four significant clusters.

Categories	Clusters			
	1	2	3	4
Role-playing with a simulated patient	yes	no	yes	no
Watching videos showing the most common mistakes	no	yes	yes	no
Watching videos showing the best ways to DBN	no	yes	yes	no
Lectures	no	no	yes	no
Participation in training led by a psychologist who provides ongoing feedback	yes	yes	yes	no
Independent literature study	no	no	yes	yes
*n*	150	132	27	12
(%)	46.7	41.12	8.41	3.74

## Data Availability

The datasets used and/or analyzed during the current study are available from the corresponding author on reasonable request.
